# Botulism Type E Outbreak Associated with Eating a Beached Whale, Alaska

**DOI:** 10.3201/eid1009.040131

**Published:** 2004-09

**Authors:** Joseph B. McLaughlin, Jeremy Sobel, Tracey Lynn, Elizabeth Funk, John P. Middaugh

**Affiliations:** * Centers for Disease Control and Prevention, Atlanta, Georgia, USA;; † United States Department of Agriculture, Fort Collins, Colorado, USA;; ‡ Alaska Department of Health and Social Services, Anchorage, Alaska, USA

**Keywords:** Staphylococcus aureus, Europe, drug resistance, microbial, methicillin resistance, epidemiology

## Abstract

We report an outbreak of botulism that occurred in July 2002 in a group of 12 Alaskan Yu'pik Eskimos who ate blubber and skin from a beached beluga whale. Botulism death rates among Alaska Natives have declined in the last 20 years, yet incidence has increased.

The incidence of botulism in Alaska is among the highest in the world, and all cases of foodborne botulism in Alaska have been associated with eating traditional Alaska Native foods, including "fermented" foods, dried foods, seal oil, and muktuk (skin and a thin pinkish blubber layer immediately underneath the skin) from marine mammals (*1**,**2*). Botulism toxins are divided into seven types; intoxication with toxin type E is exclusively associated with eating aquatic animals. Most cases of botulism in Alaska are caused by toxin type E.

On July 12, 2002, two residents of a Yup'ik village in western Alaska found a carcass of a beached beluga whale that appeared to have died sometime that spring. They collected the tail fluke for consumption, cut it into pieces, and put the pieces in sealable plastic bags. Portions were refrigerated and distributed to family and friends. From July 13 to July 15, a total of 14 persons ate some of the raw muktuk. On July 17, a physician from western Alaska reported three suspected cases of botulism from this village; all patients had eaten the muktuk. The Alaska Department of Health and Social Services began an immediate investigation to ensure proper treatment of the ill persons, identify and interview other persons exposed to the implicated food, and obtain clinical and food samples for laboratory testing.

## The Study

We sought to identify and interview every person who ate the muktuk; all exposed persons identified were evaluated by a physician or nurse practitioner for signs or symptoms of botulism. Exposed persons with signs or symptoms compatible with foodborne botulism were referred to the regional hospital for further evaluation and treatment, if necessary. A case of foodborne botulism was defined as a clinically compatible illness (Figure) with symmetric descending flaccid paralysis of motor and autonomic nerves in a person who had eaten the muktuk. Serum, stool, and gastric specimens from case-patients and leftover muktuk were collected and submitted to the Centers for Disease Control and Prevention (CDC) National Botulism Surveillance and Reference Laboratory for botulinum toxin detection, using the standard mouse bioassay (*1*).

**Figure:**
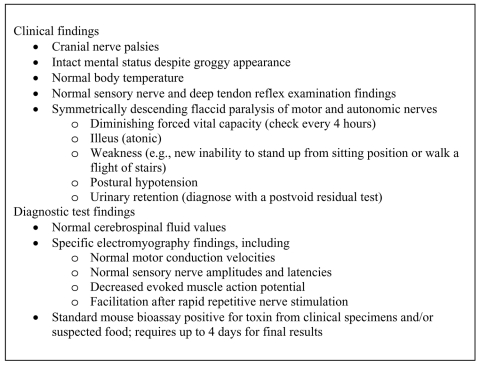
Clinical and laboratory findings of foodborne botulism.

Of 14 persons identified who ate the muktuk, 8 (57%) had illness that met the case definition. Three of the eight patients were male; the median age was 73 years (range 13–83 years). The median incubation period was 24 hours (range 12–72 hours). Signs and symptoms are shown in the Table. Five (63%) patients were hospitalized, four (50%) received types AB and E antitoxin a median of 30 hours (range 24–60 hours) after symptom onset, two (25%) required mechanical ventilation, and all survived. Both persons who required mechanical ventilation received antitoxin. Attending clinicians decided not to give antitoxin to four of the patients with milder illness because of the potential risk for adverse side effects from horse serum antitoxin.

**Table:** Signs and symptoms of eight case-patients from a botulism outbreak associated with eating a beached whale, western Alaska, July 2002

Sequelae	No. (%)
Gastrointestinal symptoms	
Abdominal pain	5 (63)
Constipation	5 (63)
Diarrhea	4 (50)
Nausea or vomiting	7 (88)
Neurologic symptoms	
Blurred vision	5 (63)
Diplopia	1 (13)
Dry mouth	7 (88)
Dysphagia	6 (75)
Dysarthria	4 (50)
Shortness of breath	5 (63)
Other symptoms	
Throat pain	3 (38)
Dizziness	6 (75)
Neurologic signs	
Hoarse voice	5 (63)
Ptosis	2 (25)
Pupils fixed and dilated	5 (63)
Urinary retention	1 (13)
Weakness	8 (100)
Other signs	
Bradycardia^a^	4 (50)
Hypotension^b^	6 (75)

^a^Heart rate <60 beats per minute. ^b^Systolic blood pressure <100 mm Hg.

Three stool, three gastric fluid, and seven serum samples from the eight patients and seven samples of whale muktuk were tested for botulinum toxin. The mean sample collection interval for serum was 3 days after exposure (range 1–5 days); for stool and gastric fluid, the mean interval was 4 days after exposure (range 3–6 days). The diagnostic laboratory received all laboratory specimens on July 26, and results were reported on August 1. Type E toxin was detected in a stool sample from one patient. This stool sample was collected on day 5 after exposure and received for testing 7 days later. All other clinical specimens were negative for botulinum toxin. All seven samples of muktuk were positive for type E botulinum toxin.

## Conclusions

An outbreak of botulism type E affected 8 of 14 Alaska Natives who ate muktuk harvested from a dead beached whale found on the remote Alaska Bering Sea littoral. Illness was promptly diagnosed and antitoxin administered. Although the median serum, stool, and gastric fluid sample collection times were within 4 days of illness onset, and all muktuk samples tested positive for toxin type E, only 1 of the 13 clinical samples from case-patients yielded positive results for toxin with the standard mouse bioassay. Both the limited sensitivity of the mouse bioassay for botulinum toxin detection in clinical specimens, as seen in this outbreak, and the fact that the test requires up to 4 days for final results demonstrate that clinicians should not wait for laboratory confirmation to make diagnostic and clinical treatment decisions.

Almost half of the cases of all types of foodborne botulism in the United States occur in Alaska, which has 0.2% of the national population. From 1990 to 2000, a total of 97 cases of botulism type E were reported in the United States; 91 (92%) occurred in Alaska. Alaska Native death rates from botulism have dropped during recent decades. Arctic explorers and whalers described deaths of entire Alaska Native families who ate whale meat (*3*). Before 1961, the botulism case-fatality rate among Alaska Natives was nearly 50%; from 1967 to 1974, it declined to 9% (*4*). From 1990 to 2000, the case-fatality rate averaged 3%, lower than that of the other 49 states. This reduction is due to several factors. First, public health efforts have educated the population and clinicians serving it about prevention, signs, symptoms, and the need for immediate treatment of botulism. Second, immediate evacuation of rural patients to modern regional hospitals, often by small aircraft, is routinely practiced. Third, trivalent botulinum antitoxin (anti-A, B, and E) is stocked in most rural hospitals so it is immediately available for treatment when clinically indicated (*5*).

Type E toxin is responsible for >85% of all botulism cases in Alaska because many traditional Alaska Native foods, including salmon heads, whale blubber, seal flesh and oil, and fish eggs are prepared by fermentation under conditions that may favor germination and vegetation of toxin type E–producing *Clostridium botulinum* (*4**,**6*). Eating blubber from whale carcasses as described in this outbreak is in accordance with tradition; however, storing blubber in airtight sealable plastic bags, which can create an anaerobic environment, is a modern development. The use of airtight containers to store and ferment traditional foods is theorized to be at least partly responsible for the increase in incidence of foodborne botulism in Alaska from 1970 to 1997 (*5*).

In his 1963 review of botulism type E, Dolman suggested a logical solution to the problem of botulism in the Arctic when he stated that "Public health educational efforts based on a proper understanding of the dangers involved can do much to reduce them by advocating relevant sanitary precautions. Besides, merely drawing attention to these hazards will accelerate the march of acculturation and thus eventually render [traditional Alaska Native foods] unpopular (*6*)." Since that time, however, anthropologic and paleontologic studies have found negative health effects associated with rapid cultural transformation (*7*), and studies have found that rapid changes from subsistence diets to affluent (Western) diets have been associated with increased incidence of coronary heart disease, obesity, diabetes mellitus, and cancer among Alaska Natives (*8**–**11*). In addition, eating traditional foods can form part of the cultural identity of groups that are in cultural transition and therefore may be perpetuated as a link to the past (*2*). To that end, state and federal public health officials have invested considerable effort in promoting intake of traditional foods among Alaska Natives, while defining safer methods for food storage and preparation (*5**,**12*).

Because botulism may have nonspecific symptoms (e.g., abdominal cramping, diarrhea, vomiting), rapid diagnosis can be difficult, even in Alaska, where the index of suspicion is comparatively elevated. However, some specific signs and symptoms (e.g., descending paralysis) may be detectable early on and are virtually diagnostic (Figure). The clinical signs and symptoms of botulism from toxin types A, B, and E are similar; however, a clinical comparison in the United States from 1975 to 1988 showed that patients with illness from type E toxin have slightly shorter incubation periods and less frequently required intubation (39% with type E, 52% with type B, and 67% with type A) (*13*). Additional current reports on botulism, including the epidemiology, diagnosis, therapy, outbreak response, and reference laboratories have been recently published (*1**,**14*).

In conclusion, clinicians confronted with a suspected botulism case should review the clinical diagnostic criteria and not rely on the mouse bioassay for toxin detection to guide clinical decision-making. In addition, although public health efforts and clinical improvements have drastically reduced botulism death rates among Alaska Natives, the average annual incidence of foodborne botulism in Alaska since 1970 is substantially higher than it was before 1970, possibly, in part, because of modern influences, such as availability of plastic, sealable containers. Finally, because of the numerous beneficial health (not to mention cultural) effects of traditional food consumption, we recommend that Alaska Native leaders continue to promote traditional food consumption among their people, while educating them about the potential hazards of improper storage and preparation (*12*).
